# Visually perceived depth from single-dot circular trajectories

**DOI:** 10.1038/s41598-025-33226-w

**Published:** 2025-12-18

**Authors:** Leo Poom, Fatemeh Roshaniasl, Mikael Fahlström

**Affiliations:** Department of psychology, Division of perception and cognition, Uppsala university, Uppsala, Sweden

**Keywords:** Visual perception, Depth from motion, Depth perception, Structure from motion, Simplicity, View-from-above bias, Neuroscience, Psychology, Psychology

## Abstract

This study investigates the perception of three-dimensional depth elicited by a single dot traversing an oblique projection of circular motion within the frontal plane. We show that this minimal motion stimulus evokes vivid depth experiences comparable to those from two-dot relative motion, demonstrating that speed variations of a single element alone enable 3D space perception. In addition, perspective projection which is an important depth-cue for other stimuli, for example resulting in linear perspective and texture gradients in stills, do not contribute much compared to parallel projection for our stimuli. The results also demonstrate a robust view-from-above perceptual bias, indicating that ecological priors influence depth disambiguation. These effects are discussed using Gestalt principles of simplicity and Bayesian models of perception. Together, these findings enrich our understanding of motion-based depth perception and highlight novel mechanisms by which the visual system reconstructs depth from minimal cues.

**Supplementary Information:**

The online version contains supplementary material available at 10.1038/s41598-025-33226-w.

## Introduction

One of the central challenges in visual perception research is understanding how the brain transforms flat, two-dimensional retinal images into vivid three-dimensional experiences. To investigate this, studies often simplify visual stimuli to isolate specific depth cues. Remarkably, even minimal inputs, such as two or three dots in relative motion, can evoke compelling impressions of depth and 3D structure^1,2^. The visual system is also capable of extracting human form and action from sparse displays, exemplified by point-light biological motion^3^. Classic demonstrations include rigid structure-from-motion (SFM), where dots on opposite sides of a rotating object move in contrasting directions^4^, and motion transparency, in which surfaces are perceived as distinct depth layers when two sets of dots move oppositely^5,6^. Depth and surface slant further emerge from motion parallax^7^, reminiscent of the shifting landscape viewed from a train window. These phenomena all rely on relative motion among simultaneously visible elements. Yet, one potential cue has received surprisingly little attention: variations in the speed of a single moving dot. Here, we investigate how such speed variations contribute to the perception of visual space.

Johansson anecdotally observed that harmonic motion is often perceived as circular motion in depth when viewed obliquely^8^. His 1971 demonstration^9^ (see at 1:58 Johansson: Motion Perception part 2) illustrates this: the motion can be interpreted either as sinusoidal speed variation in 2D or as the projection of uniform rotation in depth. Johansson noted a strong preference for the 3D interpretation. When two dots move in opposite phase (180° apart), they are perceived as connected by a rigid rod rotating in depth^10^, the simplest case of rigid SFM.

**Fig. 1:**
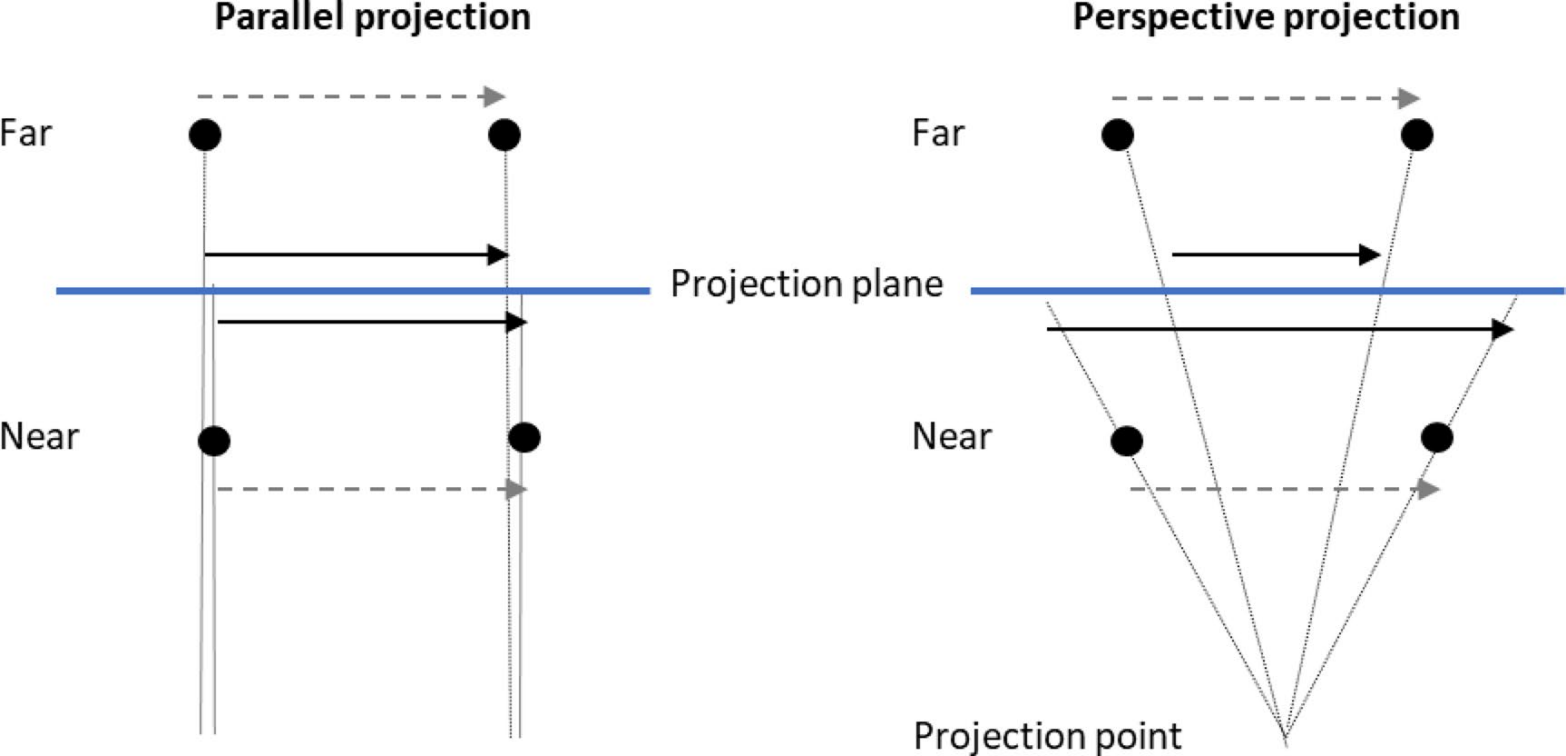
Schematic illustration of parallel and perspective projection as seen from above. The black dots represent objects moving from left to right at far and near depths relatively the projection plane. With parallel projection the traversed distance on the projection plane is not dependent on the distance in depth. With perspective projection, the projected distance traversed by the far object is shrunken and its speed is slowed down, and the distance travelled by the near object is magnified and its speed is increased. In parallel projection, the projection point is at an infinite distance from the projection plane.

Even a single dot moving along a straight path can be perceived as moving in depth if its speed varies in a way consistent with perspective projection^11^. In frontal images, near objects appear to move faster than distant ones, despite having identical distal speeds (Fig. 1). An accelerating object in the image plane may thus be interpreted as moving in depth. This aligns with the minimum principle, also known as the gestalt rule of simplicity, which favours interpretations involving the least change, such as constant speed in depth, over more complex alternatives such as one involving speed variations in the frontal plane. Johansson argued that harmonic motion in the frontal plane exemplifies this principle^8^, suggesting that speed variations in the image are interpreted as depth motion with stable speed. However, he did not empirically test this claim. He also proposed that perspective projection, rather than parallel projection, yields more veridical depth perception^10^.

**Fig. 2:**
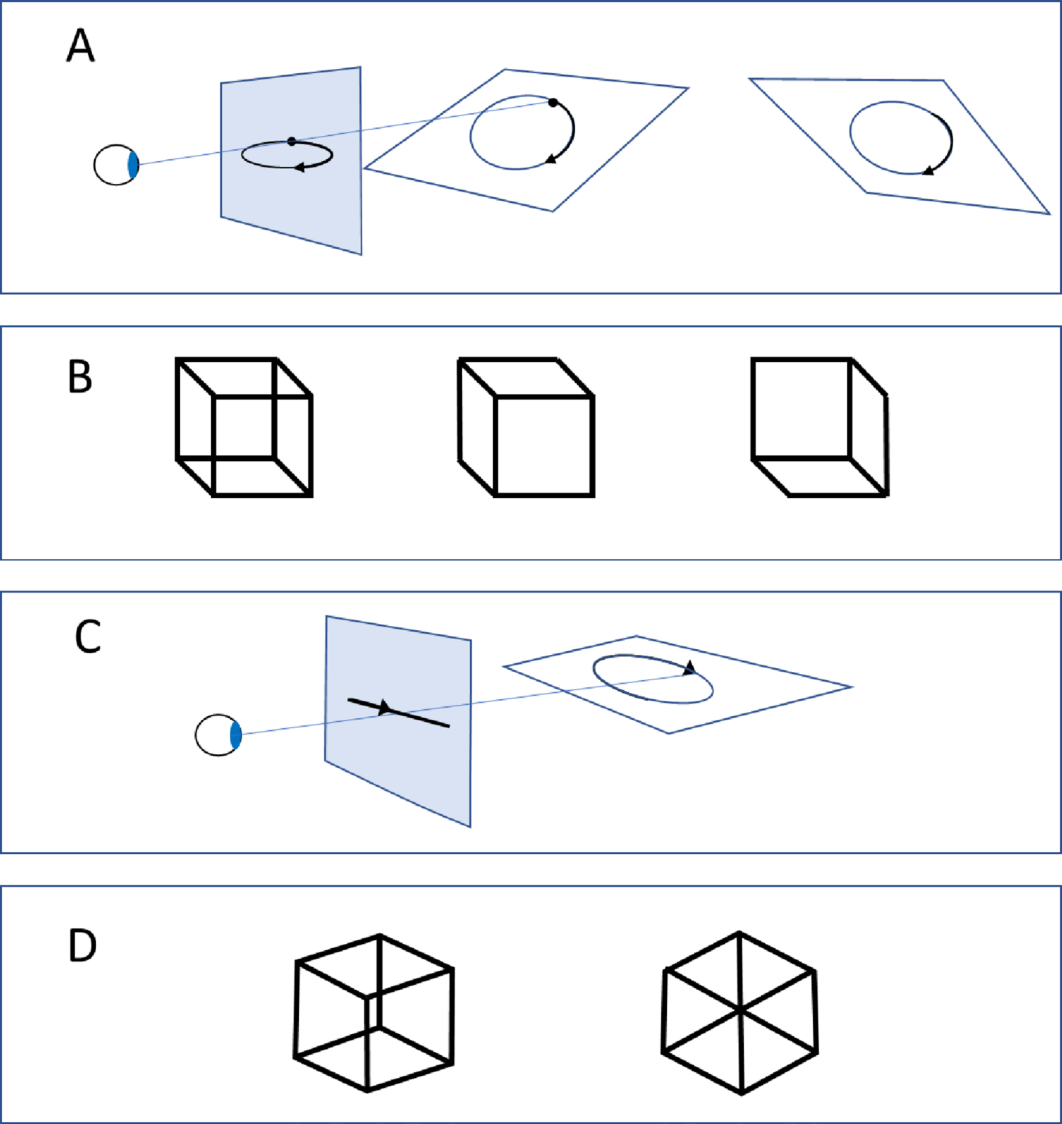
Examples of depth ambiguities and accidental viewpoints. (**A**) A circular motion in depth perceived from an oblique angle result in elliptic motion on the projection plane, which can be perceived as elliptic motion aligned with the projection plane, or circular motion in depth. The perceived motion in depth is ambiguous and may be interpreted with a view from above or from-below. (**B**) Likewise, the more familiar Necker cube is preferentially perceived in a view from above position, as in the middle, rather than from beneath. (**C**) An accidental, non-generic, viewpoint of a circular motion from the side results in a motion trajectory along a straight line on the projection plane. (**D**) A similarly generic and non-generic viewpoint of a Necker cube, where a slightly different viewpoint away from the non-generic abruptly changes the appearance from a 2D geometric patterns to a 3D cube.

In addition to the minimum principle, prior experience or past occurrences shapes perception. These two principles are often indistinguishable since the minimum principle and occurrences often arrive at the same conclusion in natural settings^12^. One such prior, that can be used to distinguish the minimum principle and influences of prior experiences, is the tendency to perceive objects as moving along the ground plane under gravity, leading to a view-from-above bias. Johansson acknowledged that circular trajectories could be perceived as slanted forward (from above) or backward (from below) depending on viewpoint (Fig. 2A)^10^, though he did not propose a systematic bias. However, the view-from-above bias is known to affect brightness and size illusions^13^, depth relations in SFM^14^, point-light walker disambiguation^15^, and Necker cube perception (Fig. 2B)^16,17^. For brief exposures, the view-from-above bias in perceiving Necker cubes is nearly universal, though it diminishes over time^18^. Ambiguous SFM slants are similarly perceived as backward slants, consistent with this bias^14,19^. Notably, prolonged exposure to microgravity reduces both the view-from-above bias and the biological motion inversion effect providing evidence for its experiential basis^20,21^. It is expected that the view-from-above bias also influence circularly perceived trajectories to be perceived from above rather than from beneath.

Another perceptual variable shaped by experience is the distinction between generic and non-generic viewpoints. Generic viewpoints yield stable interpretations across small changes, while non-generic viewpoints produce abrupt perceptual shifts. Oblique views of circular motion, resulting in elliptical retinal orbits, are generic. In contrast, side views produce linear back-and-forth motion and are non-generic (Fig. 2C). The Necker cube illustrates this as well: generic viewpoints yield stable cube perception across small shifts (Fig. 2D). Thus, common perceptual biases guide both motion-defined and static stimuli.

### Aims

Here we investigated 3D space-from-motion-perception, particularly if obliquely viewed circular motion of a single dot can result in perceived depth as vivid as the motion of two dots moving coherently around the circle. In theories that attempt to explain how the visual system recover 3D structure based solely on motion cues in a two-dimensional visual field it is typically assumed that depth is processed from input signals consisting of the relative motions between two or more dots. If the vividness of depth from a single moving dot is comparable to the vividness of depth from two dots in relative motion along the same track, then such assumptions are invalidated. The vividness of depth from the circular motion of a single dot was therefore compared to an identical motion with two-dots. Previously, vivid impressions of depth have been found from displays with only two dots moving in straight paths relatively each other^2^, and from accelerated motion of a single dot along a straight path^11^. We also investigated whether a simulated view-from-above compared to a view from below influence the perceived depth, and if the influence of perspective projection compared to parallel projection influence the vividness. The independent variables were the number of dots (one or two), projection type (perspective projection and parallel projection), and the simulated viewpoint from below (−10°), from above (+ 10°), and a side view (0°). The dependent variables were the estimated vividness of depth impression, and in trials where depth was perceived the perceived sign of depth was reported (a measure of the view-from-above bias).

**Fig. 3:**
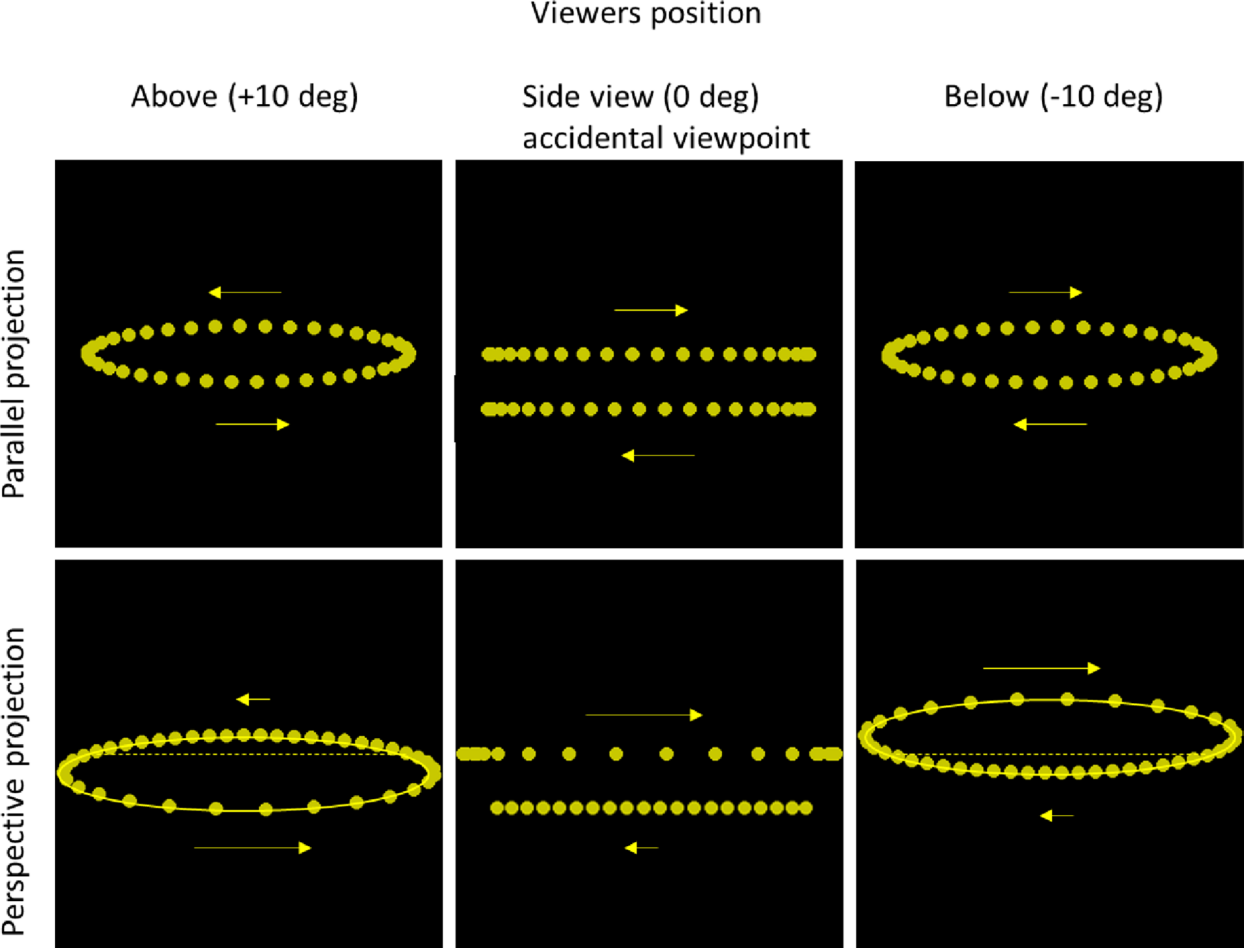
The motion paths of the stimuli. Shown are overlaid snapshots of every 3rd frame in the motion sequence with one dot, viewed from + 10°(above), 0° side view, and − 10° (below) the simulated circular motion (parallel and perspective projections). In the 0° view, the simulated front and back portions of the motion path is separated for illustrative purposes. Arrows show the velocity vectors of the motion with rightward motion corresponding to the simulated near side. The horizontal dotted lines for the perspective projection represent the diameter of the circular path, crossing its centre. Due to the perspective projection, the diameter is not aligned with the major axis of the elliptic path. At oblique angles of the circular motion, both the parallel and the perspective projection results in elliptic motion paths (ellipses results from both conic and cylindric intersections).

Figure 3 shows the stimulus conditions as sequential positions for each 3rd frame of the one-dot motion sequence. Three viewpoints were simulated: Two generic oblique viewpoints, view-from-above (+ 10°), a view-from-below (−10°), and a non-generic side view (0°). Dots moved counter-clockwise in the + 10° condition, and clockwise in the − 10° condition. One and two-dot motions were simulated to investigate if relative motion cues are crucial for depth vividness from motion. In the two-dot conditions the dots moved relatively each other at opposite sides of the circle (180° apart). Perspective projection and parallel projection were used to display all the motion sequences. Note that in parallel projection, the simulated view from above and from below differ only with respect to the opposite motion direction (counter clockwise and clockwise), but are otherwise identical.

The three simulated viewpoints can be described as two generic and one non-generic (or accidental) viewing positions. The generic positions were the two oblique views (from above and from below). The non-generic perspective was the path seen in a side view, where the dots moved back and forth along a straight line. In the parallel projection condition the viewing perspective is ambiguous, whereas with perspective projection there is a congruent condition where the perspective projection is consistent with a view-from-above. This is illustrated in the leftmost perspective projection in Fig. 3 where the dot moves with the highest speed in the lower part of its path. In the incongruent condition, illustrated in the rightmost perspective projection in Fig. 3, the dot moves with the highest speed in the upper part of its path. The speed variation in this incongruent condition is inconsistent with a view from above since the simplicity principle instead suggest a view from below. From these conditions it can be determined if the speed difference due to perspective projection in line with the simplicity principle, or the view-from-above preference in line with the prior experience of a ground level (a view-from-above bias) is dominant in interpretating these stimuli.

Supplementary Videos S1–S6 present demonstrations of all one-dot stimuli under parallel and perspective projection at viewing positions of + 10°, 0°, and − 10°. Parallel projection is shown in Supplementary Videos S1–S3, while perspective projection is shown in Supplementary Videos S4–S6. Supplementary Videos S7–S12 illustrate the two-dot conditions, with parallel projection in Supplementary Videos S7–S9 and perspective projection in Supplementary Videos S10–S12.

In the Experiment participants viewed these stimuli presented on a computer screen. Binocular disparity and vergence motion provide information that the screen is flat, and monocular viewing provides such information by accommodation and small head or eye movements relatively the screen. To minimize the influence of such cues, the screen was viewed binocularly through a large collimator lens (the same lens as used previously at this lab^1,2,11^. The collimator sets the viewing distance to infinity which remove information of both binocular and monocular cues that inform the observer of a flat screen, and increases the perceived depth from other depth cues^22^.

## Results

The freely available statistical JASP software^23^ was used to calculate frequentist p-values (with an α-level of 0.05). When possible, also Bayes factors (BF) were calculated to compare the likelihood of two competing hypotheses. BF_10_ is the ratio of the likelihood of the observed data under H_1_ to the likelihood of the observed data under H_0_ (BF_01_ is the inverse ratio). As a guideline, it has been suggested that 1 < BF_10_ ≤ 3 is considered as anecdotal evidence, 3 < BF_10_ ≤ 10 is moderate evidence, 10 < BF_10_ ≤ 30 is strong evidence, 30 < BF_10_ ≤ 100 is very strong, and beyond that extremely strong evidence^24^.

### Depth vividness

**Fig. 4:**
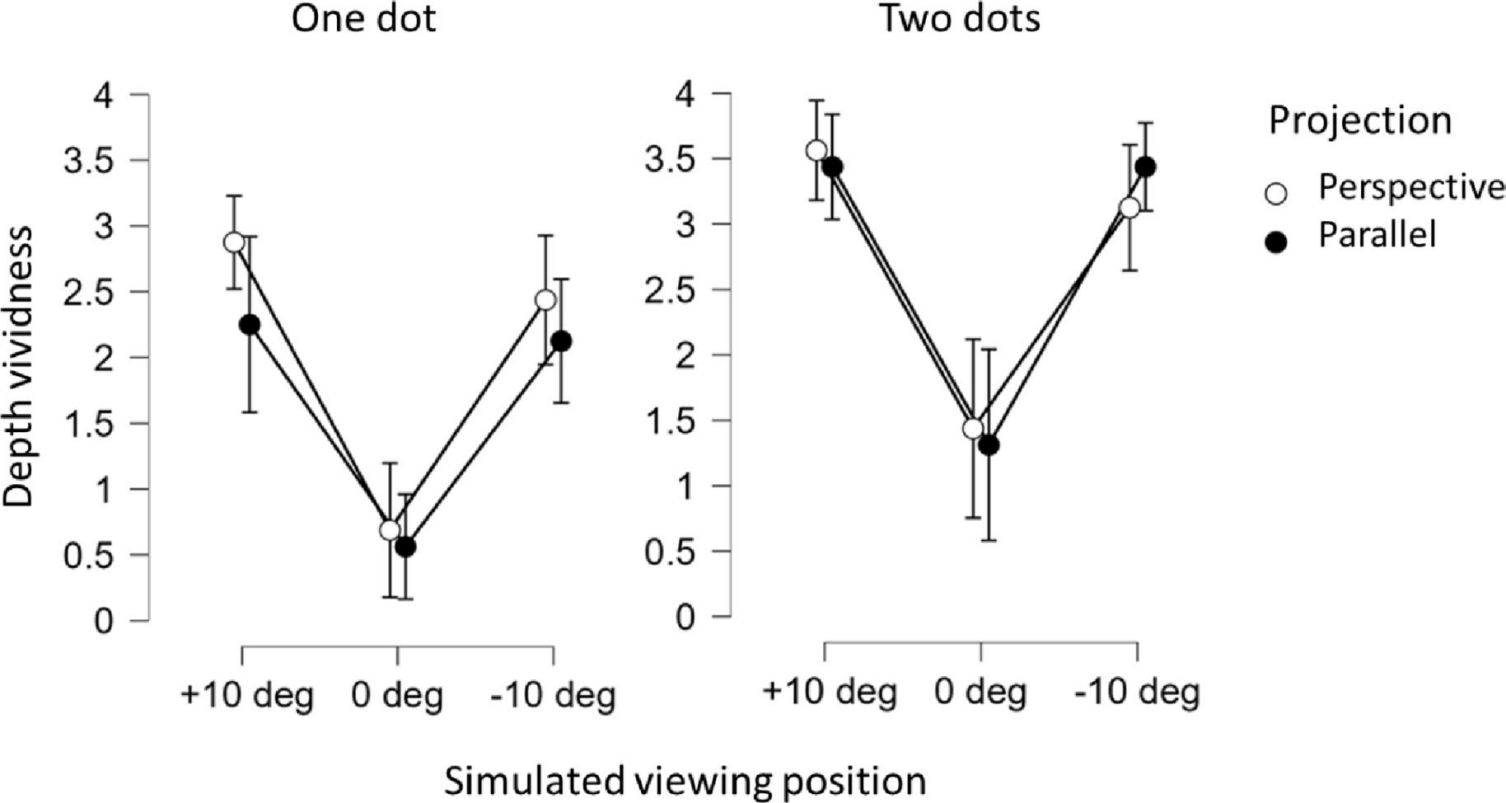
The mean vividness of depth estimates, resulting from the one and two dot circular motions with simulated projections from generic oblique angles +/−10°, and the non-generic side view at 0°. Perspective projection and parallel projection are separately displayed along with the 95% CI.

Figure 4 shows the average individual judgments of the vividness of depth impressions in the different stimulus conditions: one- and two-dot motions, perspective projection and parallel projection, and the 10º view from above, the accidental viewpoint at 0º, and the 10º view-from-below. It is apparent that the accidental viewpoint results in a weak impression of perceived depth, whereas the generic viewpoints (+/−10º) results in much stronger perceived depth.

### Simulated viewing position

Possible influences of simulated viewing position (−10°, 0°, + 10°) on depth vividness were analysed by the frequentist non-parametric Friedman one-way repeated measure ANOVA applied to the depth vividness estimates. This was done separately for the one and two dot conditions, and the two projection conditions. In the single dot and two-dot conditions the simulated viewing position of both perspective and parallel projection has a statistically significant main effect on perceived depth vividness: 19.6 < χ^2^(2) < 26.7, all *p* < 0.001.

The Bonferroni-corrected Conover post-hoc test for pairwise comparisons showed that vividness of depth in the single-dot and two-dot conditions, as well as in the projection conditions, is different between the 0° and + 10°, and between the 0° and − 10° simulated viewing positions (all p´s < 0.001), Pairwise comparisons between the + 10° and − 10° viewing positions showed that no significant differences were obtained (0.16 < *p* < 1.0).

### Two dots vs. one dot

The influence of the number of dots on depth vividness was analysed by applying the Friedman ANOVA again. When perspective projection was used two dots in the + 10° viewing position resulted in stronger depth vividness than for one dot: χ^2^(1) = 7.0, *p* = 0.008. In parallel projection the + 10° viewing position, two dots resulted in stronger depth vividness than for one dot: χ^2^(1) = 6.4, *p* = 0.011, as did two dots in the − 10° viewing angle: χ^2^(1) = 13.0, *p* < 0.001. Other differences between the one and two-dot conditions were not statistically significant (1.92 < χ^2^(1) < 2.78, 0.12 < *p* < 0.096).

### Projection type

Remarkably, no significant differences between the two projection types (perspective and parallel projection) were obtained in any of the three simulated viewing positions in any of the one-dot or two-dot conditions (0 < χ^2^(1) < 2.8, 0.096 < *p* < 1).

### View-from-above bias

We also asked the observers to report where in the motion sequence the dots appeared at the nearest position. These reports were used to investigate a if the ambiguous depth of the motion path was preferentially perceived as viewed from above rather than from below. The perspective projection results in a speed difference for the opposite motion directions as the dot passes at its nearest and farthest simulated distance. In the + 10° viewing position, the speed difference between the upmost and downmost portions of the motion path was congruent with a view from above perspective, whereas the − 10° viewing position it was incongruent. In the two-dot condition this creates a speed parallax between the concurrently presented dots, which is a well-known cue for perceiving near-far relations from relative motion where the faster features are perceived as closer. In perspective projection congruent conditions occur when the speed difference aligns with the view-from-above bias, whereas in incongruent conditions the speed difference is in conflict with the view-from-above-bias.

**Fig. 5:**
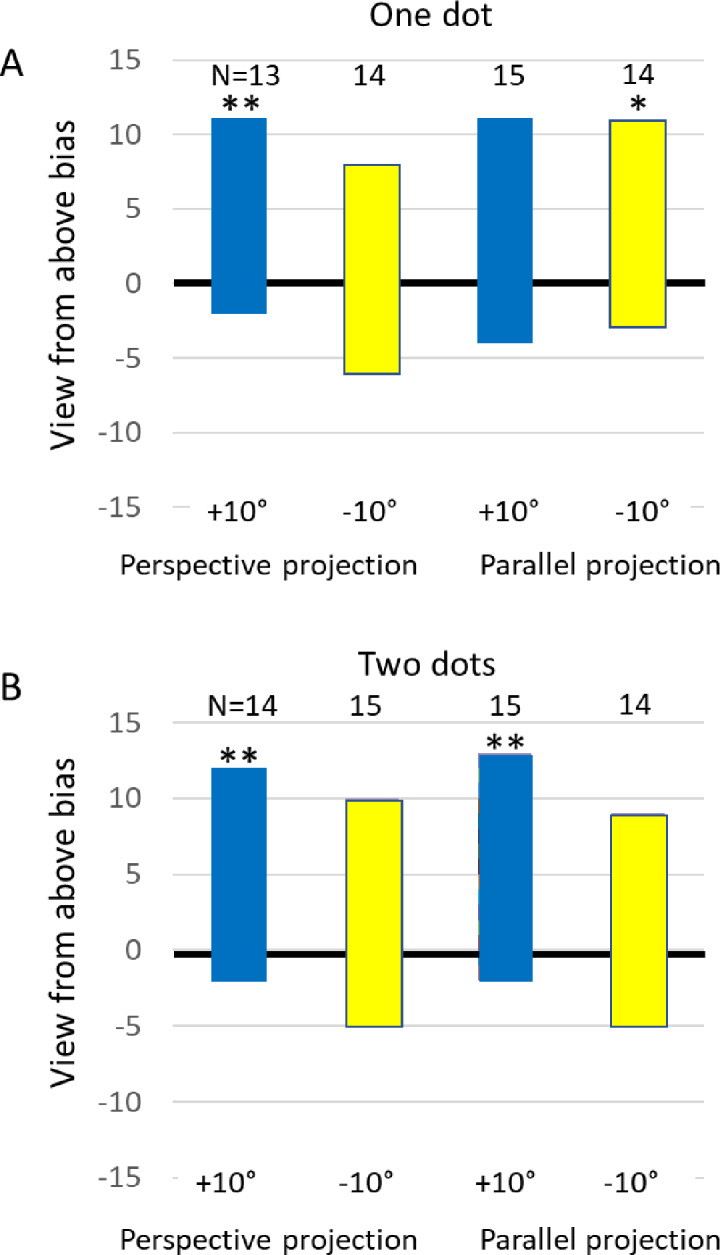
The view-from-above bias (VFA). Positive scores on the vertical axis are the number of reported depths consistent with a view-from-above. Negative scores are the number of reports inconsistent with a view-from-below. Separately presented in one dot (**A**) and two dot conditions (**B**). The total number of reports are shown above each bar. Responses not interpretable as view-from-above or view-from-below perspectives were excluded (see text). Moderate evidence from Bayes factors for a VFA-bias is indicated by *, and strong evidence is indicated by **.

Figure 5A and B show the view-from-above bias in one- and two-dot conditions, which reflects perceived depth consistent with viewing the motion path from above. Responses from 13 to 15 participants who perceived the nearest motion as either leftward or rightward were interpretable as view-from-above or view-from-below perspectives. In contrast, responses indicating that the nearest part of the path was in the middle were not interpretable and were therefore excluded from the analyses. No preference should result equal number of reports of view from above as from below. Proportions exceeding 0.5 support a view-from-above bias, as confirmed through both Bayesian and frequentist binomial tests. When perspective projection was consistent with a view from above, the Bayes factors provided strong support that a view from above was indeed preferred for the single and two-dot motion conditions with + 10° simulated point of view (BF_10_ = 15 and 24, *p* = 0.011 and 0.006 respectively). More interesting is the result that, in the parallel projection condition where the speed did not inform about relative depth, evidence for a view-from-above bias was still obtained for the two-dot motion condition with + 10° simulated point of view (BF_10_ = 39 which is considered as strong support, *p* = 0.004), and from the parallel projection of the single dot motion with a simulated − 10° viewpoint (BF_10_ = 5.9 which is considered as moderate evidence, *p* = 0.029). For the other conditions, the BF_10_ ranged between 0.5 and 2.5 which is considered inconclusive evidence for any preference, and p-values ranged between 0.059 and 0.4. Note that if the simplicity principle ruled, then the perspective projected − 10° viewpoint should be perceived as such, but this is not the case in any condition. Bayesian A/B tests showed that within each projection style and the one and two-dot conditions, the differences between the + 10° and − 10° projection angles were (at most) anecdotal (0.93 < BF_10_ < 2.2).

The non-generic viewing position at 0°, which is un-informative about any ground-level constraint, provided a weak or absent perception of depth. Therefore, we obtained only a few reports of which motion direction along the path, left or right, resulted in perceiving the dots as passing at its nearest position (8 in the perspective projection and 4 in the parallel projection in one-dot conditions, and 10 versus 7 in the corresponding two-dot condition). The Bayesian A/B test in the one-dot condition provided moderate evidence for that the dot was more frequently reported as nearest when passing at its fastest speed than when passing at its lower speed (BF_10_ = 7.0). In the other non-generic conditions, we obtained no reliable evidence for the influence of speed as a result from perspective projection (0.6 < BF_10_ < 1.3). The failure of the two-dot non-generic condition to produce perceived depth, or a reliable depth sign, may be due to the appearance of collisions between the dots as they passed in opposite directions along the straight paths.

## Discussion

We showed that vivid depth is perceived from a circular motion of a single dot obliquely projected upon a 2D surface, and appears only slightly less vivid than if two dots are moving along the same path. This is remarkable since theories that attempt to explain how the visual system recovers 3D space from the motion patterns in 2D retinal images, assume that concurrent relative motion is crucial^25,26^. Although perspective projection contributes with more information as compared to parallel projection, and could disambiguate the near far relations, it did not significantly increase the vividness of depth in our sample. When a single dot moves around the circle, depth from direction and speed comparisons between the far and near positions requires integration of motion over time. In contrast to the oblique generic +/−10° viewing positions, the accidental, or non-generic viewpoint conditions resulted in low vividness of perceived depth, even when perspective projection was used and the left and rightward motion differed in speed. This is consistent with other static stimuli, such as the Necker cube where accidental view-points breaks the appearance of a 3D cube and results in an appearance of a 2D geometrical pattern. Mostly, the non-generic view point of the moving dots was similarly perceived as moving back and forth along a straight line in a frontal 2D plane. In the two-dot parallel projection condition, one of the authors frequently perceived the dots as colliding and abruptly reverse direction, and could trigger this percept at will, suggesting top-down process.

Although perspective projection is used by artists to create a sense of depth and realism in pictures and may contribute to 3D shape perception, it did not increase the vividness of perceived depth in our motion stimuli. It is likely that the perspective projection contributed with small effect sizes that could be detected in a larger sample. Perspective projection added to the view-from-above bias in congruent conditions when the perspective was consistent with a view from above, but it failed to counteract this bias in incongruent conditions. A view-from-above bias was also observed in the parallel projection conditions. With a larger sample it is possible that the view-from-above bias could be reliably detected in all our stimulus conditions. The view-from-above bias influence size and brightness illusions^13^, resolve various ambiguities, such as the Necker cube^16,17^, and ambiguous biological motion point-light-walkers^15^. In addition, ambiguities may be resolved by direction of gaze as shown for Necker cubes^27^, SFM^28^, and for point-light walkers, where a preference to direct attention to the lower part of the figure promote a preference for perceiving the knees as bulging outwards, causing a convexity bias^29^. It may also be a consequence of a bias resulting from a preference of focusing on approaching people^30^. A preference in directing the gaze to the lower parts of stimuli may contribute to the view-from-above bias observed in the present study, although our data do not provide evidence for the involvement of gaze direction. Taken together, these results support the hypothesis that the ecologically rooted experience-based view-from-above bias play a critical role in resolving visual ambiguities and overrides the information obtained from perspective projection.

Small effect sizes typically require many trials to achieve reliable conclusions. In contrast, perceptual phenomena often produce large effects with low inter-individual variability, enabling robust inferences from relatively few participants and trials. This reduces concerns about learning or adaptation across repeated measures. The assumption that perceptual mechanisms are broadly shared across individuals is longstanding, as illustrated by classic Gestalt demonstrations such as Rubin’s vase and the Kanizsa triangle, compelling examples that remain influential despite lacking formal statistical validation in initial publications. Numerous similar findings, though derived from small samples, have been consistently replicated, in sharp contrast to other psychological domains where weaker effects necessitate larger samples to ensure reliability.

A potential motion-direction bias could have influenced the results: in perspective projection, the faster speed coincided with rightward motion (Fig. 3), which may have acted as a confound. However, no reliable evidence for a direction-bias was obtained from the parallel projections where there was no speed difference between leftward and rightward motion. Using the collimator screen minimized conflicting binocular depth cues such as binocular disparity and vergence which could have influenced the results. The collimator also minimized monocular cues such as motion parallax due to small head or eye movements and accommodation. This technique has been employed before^1,2,11^, and has been shown to increase visual vividness of depth from pictorial depth cues^22^. Another conflicting depth cue that was not counteracted was that an object moving in depth change size in the projection plane, expanding and contracting as the approach and recede according to Emmert’s law describing how retinal size of objects changes with its distance from the observer when using perspective projection. Here the dot size was fixed since we didn’t want to add other cues to depth than the dot motions, but this might have counteracted perceived motion in depth, and resulted in underestimations of depth vividness estimates.

The Gestalt principle of Prägnanz suggests that the brain favours the simplest motion-based depth interpretations. Bayesian models, on the other hand, argue that depth perception relies on prior knowledge and statistical regularities of the real-world. These explanations can account for the perceived depth from speed variations along elliptical paths by considering the relative likelihoods of different motion patterns such as uniform vs. variable speed, and circular vs. elliptical trajectories. However, such priors assume that we have previously encountered such elliptical projections on the retina and found out that distal circular 3D paths caused them, which lead to a circular reasoning^31^. But simplicity priors could be used as an alternative to the experience-based probability priors in Bayesian models, since experience-based priors are often indistinguishable from “simplicity-priors” as formulated by the Gestalt principles^12^. The simplicity, or minimum principle claim that “the perceptual response to a stimulus will be the one that requires the least amount of information to specify.” ^8^. The Minimum Description Length (MDL) principle is a formalized version of this idea, used to evaluate competing hypotheses about sensory input. Inspired by Kolmogorov complexity^32^, the MDL selects the perceptual interpretation that minimizes the combined length of description of the hypothesis and encoding the sensory data with that hypothesis. In this way, the brain effectively compresses complex motion information into the simplest and most stable depth representation. This principle helps explain why the visual system tends to interpret depth from varying motion speed in a way that favours smooth, non-varying motion in depth rather than speed variations, acceleration and deceleration in the frontal plane, whereas the view-from-above bias reflect an experience based prior.

What neural processes could underlie the perceived depth from accelerated and circular paths of single dots? It is known that neurons selectively sensitive to speed gradients, as arising from motion parallax^33^, and neurons signalling the near-far relations in transparency-from-motion, as seen in structure-from-spinning-motion^34^ exist in the motion-specialized middle temporal (MT) area of the visual cortex. Could low level neural units tuned to acceleration underlie the perceived depth from accelerated motion along straight paths as previously demonstrated^11^? Viewed from oblique angles, or from the side, the motion of a single dot moving in circles also produces accelerated motion as it turns back and forth along its track. Representation of depth from accelerated motion, or motion along obliquely viewed circular paths could involve distributed neural populations of activity. Functional brain imaging show that multiple brain regions, such as visual areas V1, V2, V3, V3A, and V4, specifically encode periodic movement paths in contrast to non-periodic motion or static stimuli^35^. It is known that perceived 3D structure-from-motion is facilitated by perspective projection and acceleration^36^. But although the acuity of estimated location of accelerated objects disappearing behind an occluding surface is high^37^, so far there is no evidence for the existence of neural detectors tuned to acceleration in humans^38,39^, although neurons in the pigeon brain seem to encode acceleration of stimulus motion^40^. Instead, changes in speed and direction in the frontal plane seem to be processed within a temporal window^41^. An obliquely viewed single dot in circular motion may involve a process that compare velocities over time: as the dot moves across a given position, its current speed and direction are compared with its speed and direction at a previous moment when it was at the opposite side of the circle. Such temporal summation occurs for higher-order stimulus features, such as point light biological motion displays^42^.

The primary dependent measure was operationalized as the vividness (clarity or strength) of the subjective impression of depth, rather than the perceived distance in depth. Thus, vividness is a phenomenological quality that can vary independently of metric depth judgments. This can be illustrated by the difference between stereoscopic depth and depth perceived from pictorial cues in stills, where it can be perceived that the picture is actually flat whereas at the same time the implied distance in depth is large. Stereoscopic depth, on the other hand, is experienced as very vivid compared to the large metric depths perceived from pictorial cues. Relative motions between multiple elements in a SFM display evoke perceive depth vividness comparable to stereoscopic images, and rely on common processes^25^. The speed of circular motion may influence perceived vividness of depth (as found from informal inspections). Vivid depth was perceived when a lap around the circle took 1.9 s which was the speed chosen. It corresponds to a frequency 1/1.9 ≈ 0.5 Hz of motion back and forth in depth. A previous study showed that the maximum perceived motion in depth, and perfect size constancy, occurred at size fluctuations around 2 Hz and decreased for both lower and higher frequencies^43^. It could be the case that our choice of frequency is sub-optimal in generating perceived depth for the general population. This and other boundary conditions for depth perception from the motion of a single dot is a subject for future investigations.

Our results demonstrate that even a single moving element can evoke vividly perceived depth, challenging models that rely on relative motion between multiple stimuli. By highlighting the role of temporal speed variations and ecological priors such as the view-from-above bias, this work reveals fundamental principles of spatial perception. These insights provide new constraints for computational accounts and point toward future investigations of minimal motion cues in naturalistic vision.

## Methods

### Participants

A total of 18 adult participants took part in the experiment, mostly university students. All were naïve as to the purpose of the experiment and were unfamiliar with the stimuli presented to them. Two of these were excluded from the analyses due to missing data and failure to follow instructions. The number of participants is in line with other studies targeting similar research questions and using similar methodology, and was deemed sufficient to detect robust depth perception effects^1,2,11^. Participants were compensated with a cinema ticket for their participation. The experiment took about 20 min.

### Ethics declarations

The study was conducted in accordance with the code of ethics of the World Medical Association (Declaration of Helsinki). In this study participants observed moving dots on a computer screen and reported perceived depth. These reports were the only data collected. The study involved no physical intervention or biological material taken from the participants, they were neither physically nor psychologically manipulated, and faced no risk of being harmed physically or psychologically. Participants were informed that they could withdraw at any time without losing their compensation. All data were anonymized, and no sensitive personal data was collected. Written informed consent was obtained from all participants prior to the study. According to the Swedish act concerning the Ethical Review of Research involving humans (2003:460), ethics approval is required only if the study involves physical or psychological influence, sensitive personal data, or biological sampling. As none of these conditions applied, national legislations allow not requiring approval. This was confirmed prior to the study via verbal communication with a senior representative of the Swedish Ethical Review Authority. The study was conducted at Department of psychology, Uppsala university, which concurs with this assessment.

### Stimuli and equipment

Circular motion paths in 3D space were simulated and projected on a projection plane intersecting the paths diameter. Stimuli were presented on a 23-inch Samsung monitor (the projection plane) with resolution 1929 × 1080, and 60 Hz refresh rate.

The radius of the circular motion, and the horizontal amplitude of the motion path were 5 cm on the screen in parallel projection conditions and slightly larger in perspective projection conditions (due to the expansion of the simulated near part of the motion track, as seen in Fig. 2). Dots had 2 mm radius and were yellow with RGB coordinates 200, 200, 0, and luminance 130 cd/m^2^ on a black background (0, 0, 0). The dots moved around the simulated circular path at 189°/sec., with a turn completed in 1.9 s. The movie sequence consisted of 120 frames, so dot locations were updated at 3° step positions around the path. To simulate viewing positions, dot coordinates were rigidly rotated around a horizontal axis intersecting the path’s diameter, then projected onto the projection plane.

A projection distance of 10 cm was used to simulate perspective projection, and 10 000 cm to simulate parallel projection (the distance from the projection point to the midpoint of the simulated circular motion, Fig. 1). Perspective projection, results in a speed difference of the near and far part of the simulated circular motion (Fig. 3, bottom panels). In parallel projection conditions, dot speed was identical at both the near and far segments of the motion path. In contrast, under perspective projection, the dot at the simulated near segment exhibited twice that speed, while at the far segment it moved at approximately 0.67 times the speed observed in parallel projection.

With perspective projection and the simulated + 10° oblique viewing position, the speed difference between the downmost and topmost parts of the path was congruent with a view-from-above, whereas in the − 10° viewing position, the speed difference was incongruent. In the non-generic 0° viewing position, the dots moved back and forth along a straight path, but with a higher speed when passing the near side of the simulated motion track. With parallel projection and the simulated +/−10° oblique viewing position, dots moved at equal speed at the downmost and topmost parts of the path. (Fig. 3, top panels). In the non-generic 0° viewing position, the dots moved sinusoidally back and forth along a straight path.

**Fig. 6:**
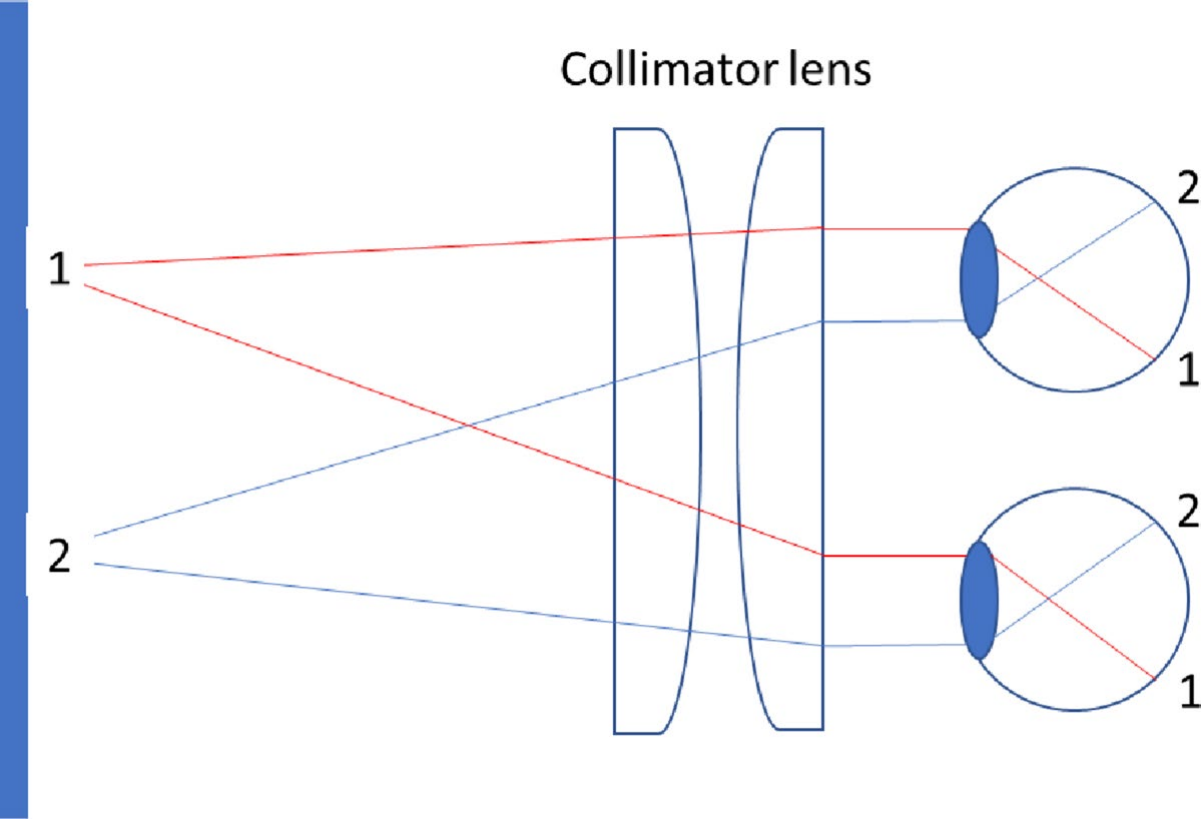
The collimator lens setup in front of the screen. The two locations on the screen project along parallel rays to the eyes. This produces identical retinal images in both eyes, rendering retinal disparity, motion parallax, and accommodation ineffective as depth cues, by places the focusing distance at infinity.

To eliminate stereo-depth cues, a collimator lens (diameter 42 cm) was used to set the apparent screen distance to infinity (Fig. 6). While binocular disparity is informative at short distances, it becomes negligible at large distances where the left and right retinal images become identical. Importantly, the absence of information from stereo-depth, as during monocular viewing, is not the same as information of absence of stereo-depth, as when looking binocularly through the collimator lens. Other advantages of using a collimator lens, as compared to monocular viewing, is the absence of monocular depth cues such as motion parallax resulting from small head or eye movements, and accommodation cues. The monitor was placed 45 cm behind the collimator lens (at its focal point), and observers viewed the stimuli from approximately 20 cm in front of the lens through an aperture in a black cloth that concealed the screen edges. The collimator lens slightly magnified the visual stimuli.

### Procedure

The procedure and method were adapted from previous similar studies investigating depth vividness from sparce motion stimuli^1,2,11^. Before the experiment, the observers were told that one or two moving dots would be presented. It was suggested that these dots might appear to move in a frontal plane in elliptic paths or motion back-and-forth without any depth, moving in depth in circular paths, or some combination of these alternatives.

Stimulus patterns within parallel and perspective projection conditions were randomized. The order of the parallel and perspective projection conditions was counterbalanced across participants: either all randomized parallel projection patterns were presented first, or the randomized perspective projection patterns were presented first. Each stimulus was presented once resulting in 12 presentations, excluding demonstrations before the experiment. Effect of learning and adaptation from repeated trials were thus minimized.

The room was darkened. Inspection time at each trial was free, which reduced time-pressure effects on depth judgment. No chin rest was used. While observing the stimuli through the collimator lens, participants were first asked whether they perceived any depth in the display. The vividness rating was zero if no depth was perceived. If they reported perceiving depth, they were asked to rate how vividly they experienced the depth. Participants were explicitly instructed to rate how clear, distinct, and compelling the depth impression felt to them, regardless of its spatial extent. This distinction was emphasized in the instructions to avoid conflating strength/vividness with perceived distance. Specifically, they were informed that a rating of 1 indicated a very subtle or weak impression of depth, whereas a rating of 4 indicated a very clear and vivid three-dimensional impression. The five-point rating scale for depth vividness increases consistency, and comparability relatively a magnitude estimation method. Discrete categories simplify decision-making and reduce intra-individual variability across trials. Unlike magnitude estimation, fixed scales are less prone to anchoring and drift, and in this context, five levels offered sufficient resolution without encouraging over-precision.

If participants perceived depth, they were also asked to report if the leftward motion was perceived closer than the rightward motion, or if the rightward was perceived closer than the leftward motion. These discrete judgments were transformed to perceived view from above or from below angles. If a rightward motion was perceived as near in conditions with a simulated viewing perspective + 10°, which is a simulated view from above, then this motion was also perceived as such. If leftward motion was perceived as near, then this circular motion in depth was perceived as viewed from below. When simulated viewing perspective was − 10°, then the relation between reported direction and viewing position was the opposite.

### Statistical analyses

Reported Bayes factors (BF) were computed using the default settings in JASP for the priors of effect size and distribution under H_1_. Unlike the p-value, which provide an index against the null hypothesis, the BF provides evidence both for and against the null hypothesis.

Since the depth vividness data are on a ordinal scale, the non-parametric Friedman one-way repeated measure ANOVA was applied to the depth vividness estimates. The frequentist two-sided p-values were calculated since no Bayesian version of this test was available. In two-level comparisons the Freedman test is identical to the sign test.

For the dichotomous estimations of perceived view-from-above in contrast to a view-from-below, we performed Bayesian and frequentist Binomial tests. The null hypothesis is that the proportions were equal (0.5), whereas the alternative hypothesis was that the proportions deviated from equal proportions, providing evidence for a bias. For the Bayesian analyses the shape of the prior distribution under the alternative hypothesis was specified by a flat prior Beta distribution, i.e., Beta(1, 1). The binomial test used to provide statistical support for the view-from-above bias calculates the exact probability of obtaining the observed results (or more extreme results) under the null hypothesis, without relying on approximations. This makes it particularly useful for small sample sizes where an approximation like the chi-square test might be inaccurate.

## Electronic Supplementary Material

Below is the link to the electronic supplementary material.

Supplementary Material 1

Supplementary Material 2

Supplementary Material 3

Supplementary Material 4

Supplementary Material 5

Supplementary Material 6

Supplementary Material 7

Supplementary Material 8

Supplementary Material 9

Supplementary Material 10

Supplementary Material 11

Supplementary Material 12

## Data Availability

The data from this study are available from the corresponding author, [LP], upon request.
